# Update on Nox function, site of action and regulation in *Botrytis cinerea*

**DOI:** 10.1186/s40694-016-0026-6

**Published:** 2016-10-07

**Authors:** Robert Marschall, Ulrike Siegmund, Joachim Burbank, Paul Tudzynski

**Affiliations:** Institut für Biologie und Biotechnologie der Pflanzen, Westfälische Wilhelms Universität, Schlossplatz 8, 48143 Münster, Germany

**Keywords:** *Botrytis cinerea*, NADPH oxidase, ROS, ER, Regulation

## Abstract

**Background:**

The production of reactive oxygen species (ROS) and a balanced redox homeostasis are essential parameters, which control the infection process of the plant pathogen *Botrytis cinerea*. The necrotrophic fungus is able to cope with the plants’ oxidative burst and even produces its own ROS to overcome the plants’ defense barrier. Major enzyme complexes, which are responsible for the production of superoxide, are NADPH oxidase (Nox) complexes. They play a central role in various growth, differentiation and pathogenic processes. However, information about their regulation and the integration in the complex signaling network of filamentous fungi is still scarce.

**Results:**

In this work, we give an update on Nox structure, function, site of action and regulation. We show that functionality of the catalytic Nox-subunits seems to be independent from their transcriptional regulation and that the membrane orientation of BcNoxA would allow electron transport inside the ER. Following previous studies, which provided evidence for distinct functions of the NoxA complex inside the ER, we highlight in this work that the N-terminus of BcNoxA is essential for these functions. Finally, we elucidate the role of BcNoxD and BcNoxB inside the ER by complementing the deletion mutants with ER bound alleles.

**Conclusions:**

This study provides a deeper analysis of the Nox complexes in *B.* *cinerea*. Besides new insights in the overall regulation of the complexes, we provide further evidence that the NoxA complex has a predominant role inside the ER, while the NoxB complex is mainly important outside the ER, likely at the plasma membrane. By considering all other putative Nox complex members, we propose a putative model, which describes the distinct complex pattern upon certain differentiation processes.

**Electronic supplementary material:**

The online version of this article (doi:10.1186/s40694-016-0026-6) contains supplementary material, which is available to authorized users.

## Background

*Botrytis cinerea* is a filamentous fungus, which is classified as second most devastating plant pathogen [10] infecting more than 500 economically important hosts [13]. It is the causative agent of the gray mold disease diminishing harvest yields of strawberries, raspberries and grapes [52]. In its lifestyle as well as in the interaction with its host, reactive oxygen species (ROS) play a central role. The molecules are produced in all cells that depend on molecular oxygen. ROS have an ambivalent role since they work as signaling molecules but also interact unspecifically with macromolecules such as DNA and lipids [4]. While plants produce ROS during the so-called “oxidative burst” as first defensive line upon contact with pathogen derived elicitors, *B. cinerea* produces ROS to trigger plant’s defense reactions, on which it depends to achieve full pathogenicity [18].

ROS are produced in highly conserved processes; they occur as mere byproducts of the respiratory chain or as results of evolved enzymatic reactions. Specialized enzymes, which are one of the major sources of ROS, are NADPH oxidase (Nox) complexes. Nox complexes are responsible for the transfer of electrons from NADPH as electron donor across membranes to molecular oxygen [27, 51]. Nox are best characterized in mammalian systems, since localization and function of Nox in these systems have been extensively studied in recent years [29]. The catalytic subunit (gp91^phox^/Nox2) is a transmembrane protein which is stabilized by the likewise membrane standing adaptor protein p22^phox^. For activation of the multi-enzyme complex cytosolic, regulatory components such as p67^phox^, p40^phox^ and p47^phox^ are recruited to the catalytic subunits.

Despite of the evolutionary distance, there are quite a few homologies between mammalian and fungal Nox complexes. Besides homologous proteins to the catalytic (gp91^phox^—NoxA/B or Nox1/2 respectively) and the regulatory subunit (p67^phox^—NoxR), more recently equivalents of the adaptor protein p22^phox^ (NoxD [25, 44]) and the scaffold protein IQGAP (Iqg1 [32]) were identified. In fungi, Nox complexes exhibit distinct functions [1, 49]. While the NoxA (Nox1) complex is controlling fruiting body formation [7, 28, 31], formation of sclerotia [14, 40, 42] and conidial anastomosis tubes (CATs) [38], virulence [23, 42, 53] and cellulose degradation [5], the NoxB (Nox2) complex is responsible for penetration of host tissue [12, 42], the production of ROS [33] and ascospore germination [7, 31]. However, the exact composition of the Nox complexes, their site of action as well as the integration in existing signaling pathways are still poorly elucidated. A major contribution for answering those questions might be recent findings that an ER locked allele of NoxA in *B. cinerea* is capable to partially restore the deletion mutants’ phenotype [44], revealing that the NoxA complex is active inside and outside of the ER.

For a further functional characterization of the NoxA complex in *B.* *cinerea*, complementation studies with an N-terminal truncated version of BcNoxA were conducted. They reveal that the N-terminus of BcNoxA is essential for a functional NoxA complex inside the ER. Moreover, we demonstrate by topology studies with the genetically encoded biosensor roGFP2 that the membrane orientation of the catalytic subunit BcNoxA would allow the production of superoxide inside the ER. In contrast to all expectations, cross-complementation studies give rise to assume that transcriptional regulation of both Nox complexes plays only a minor role during the production of sclerotia and conidial anastomosis tubes (CATs) as well as during pathogenic processes. Finally, we highlight that in contrast to the NoxA complex, the NoxB complex fulfills its predominant role outside the ER. Thus, complementation of the deletion mutant Δ*bcnoxB* with an ER retained version of the catalytic subunit revealed that BcNoxB is necessary for stress sensitivity inside the ER. Instead, all other processes such as ROS production, pathogenicity and formation of functional appressoria are putatively regulated by BcNoxB outside the ER, most likely at the plasma membrane.

## Methods

### Cultivation of *Botrytis cinerea*

*Botrytis cinerea* Pers.:Fr. [*Botryotinia fuckeliana* (de Bary)Whetzel] B05.10 was isolated from *Vitis vinifera* [6] and was used in this study as basis strain and control in all experiments. Further strains are listed in Additional file 1: Table S1.

For cultivation synthetic complete medium (CM) [37] was used. For transformation, the strains were grown on PDAB medium (Potato dextrose agar [Sigma-Aldrich Chemie, Steinheim, Germany] supplemented with 100 g/l homogenized leaves of French beans (*Phaeseolus vulgaris*). Plates were grown for 6–8 days at 20 °C under light conditions (12 h light/12 h darkness, full spectrum light) to obtain conidia. Sclerotia production was induced by incubating the strains for 3 weeks at 20 °C in darkness. For DNA preparation, the strains were grown 3–4 days at 18 °C on CM agar overlayed with Cellophane. For stress experiments, the complete medium was supplemented with osmotic, oxidative, cell wall, membrane or ionic stress. Minimal medium was prepared after Czapek Dox (20 g/l sucrose, 3 g/l NaNO_3_, 1 g/l K_2_HPO_4_, 0.5 gl KCl, 0.01 g/l FeSO_4_·7H_2_O, 0.5 g/l MgSO_4_·7H_2_O, pH 5.2).

### Generation of mutant strains

**Fig. 1:**
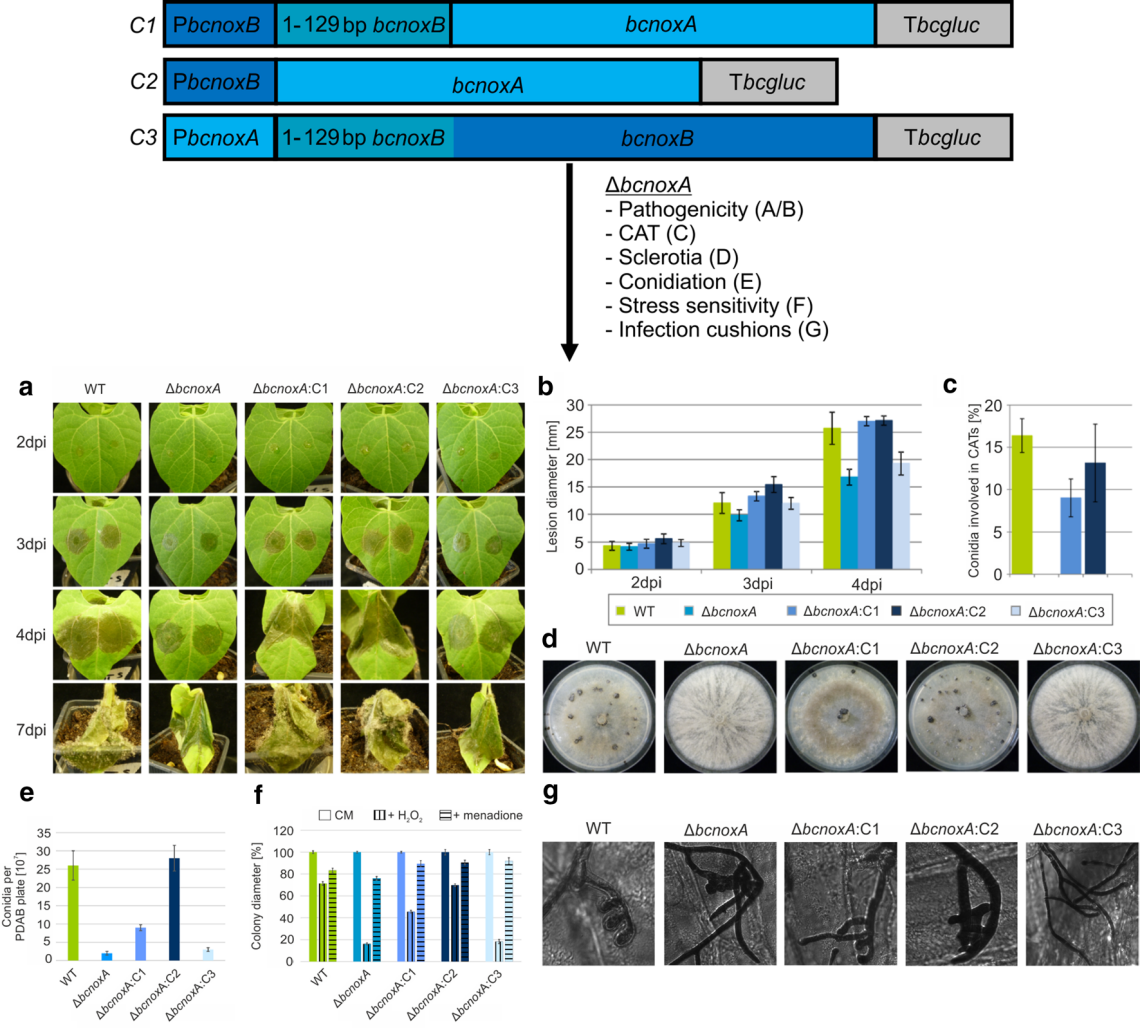
Transcriptional regulation seems to be not important for NoxA function. Three complementation constructs (C1–C3) were generated and transformed in Δ*bcnoxA*. They were integrated into the *bcniiA* locus (Additional file 1: Table S1). **a, b** Wild type like spore-mediated infection is restored in Δ*bcnoxA*:C1 and Δ*bcnoxA*:C2. Primary bean leaves were infected with 7.5 µl of conidial suspension (10^5^ conidia/ml). Lesion diameters were measured and statistically evaluated (3 bean plants/strain). Replicates showed similar results. **c** Only the mutant strain with the construct C2 is able to fully restore the formation of CATs. Conidia were inoculated on Vogel’s minimal medium for at least 18 h. Hyphal fusions were detected microscopically. For statistic evaluation, 300 spores were analyzed. **d** Restoration of wild type like sclerotia formation is mediated by the C2 construct. Agar plugs were inoculated on complete medium in constant darkness for at least 14 days. Replicates showed similar results. **e** Only Δ*bcnoxA*:C2 produces the same amount of conidiospores as the wild type. Spores were harvested from CM plates with 10 ml H_2_O and quantified in replicates. **f** Plate assays for the determination of the stress sensitivity display identical results for the wild type and the strain Δ*bcnoxA*:C2. Agar plugs were placed on CM agar and medium supplemented with H_2_O_2_ (10 mM) or menadione (500 µM). Monitoring was accomplished for 7 days (here depicted: 3 dpi). **g** Infection cushion formation is restored in the strains Δ*bcnoxA*:C1 and Δ*bcnoxA*:C2. Onion epidermal layers were inoculated with agar plugs of the respective strains. Staining of fungal hyphae was performed with lactophenol blue just before microscopy

For the generation of deletion and complementation constructs, the yeast homologous recombination system was used [9, 39]. For the cross-complementation constructs the appropriate primer were used to amplify the gene of interest with respective overlaps. Construct 1 (C1) was generated by using the primer 1/17 (Additional file 2: Table S2) as well as 2/3. The construct comprises the coding region of BcNoxA in addition to the N-terminal elongation of BcNoxB under the control of the BcNoxB promoter. For the second construct [C2 (ORF of BcNoxA under the control of the BcNoxB promoter)] the primer 1/17 and 3/4 were used. Construct 3 (ORF of BcNoxB under the control of the BcNoxA promoter) was accomplished by primer 5/18 and 6/7. The truncated version of *bcnoxA* was amplified with the primer 1/16 and integrated into the vector pNAH_OGG [39]. The truncation was accomplished by removing the putative signal peptide (Fig. 1). For topology studies, the genetic encoded biosensor roGFP2 was fused to the N- and C-terminus of *bcnoxA*. For the generation of the construct roGFP2_NoxA segments were amplified by 8/15 and 13/14; the construct NoxA_roGFP2 was generated by using the primer 9/10 and 11/12. The fusion of the HDEL motif for ER retention to *bcnoxD* and *bcnoxB* was accomplished by PCR reactions using the primer 29/30 (BcNoxD_HDEL) and 31–34 (BcNoxB_HDEL). The ER retention signal was fused to the C-terminus right in front of the stop codon. The respective constructs were cloned into the vector pNDN-OGG [39], which was digested with *Nco*I (for BcNoxD_HDEL) or *Not*I (for BcNoxB_HDEL) in advance.

After transforming *S. cerevisiae* FY834, positive transformants were selected on SD medium lacking uracil since the strain is auxotroph for this amino acid. Total DNA was isolated and transformed in *E.* *coli*. Re-isolation was done by the Nucleo spin^®^ plasmid easypure kit (Macherey–Nagel, Düren, Germany) and correct assembly was tested by sequencing.

*Botrytis cinerea* was transformed with 40–60 µg of plasmid DNA as described previously [19]. Selection was done via hygromycin (70 μg/ml of hygromycin B [Invitrogen, San Diego, USA] or nourseothricin (50 μg/ml of nourseothricin [Werner-Bioagents, Jena, Germany]). Positive transformants were purified by single spore isolation: Conidia were spread on the appropriate selective medium; afterwards, germinated conidia were picked and cultivated on new selective plates. Genomic DNA isolation was performed according to Cenis [8]. Correct and ectopic integration were checked by diagnostic PCR and Southern blot (primer 19–28 and 35–40).

### Growth and pathogenicity assays

For characterization of the different mutant strains various growth, differentiation and pathogenicity assays were performed. For testing defects in pathogenicity, French bean plants (*Phaseolus vulgaris*) were infected according to Klimpel et al. [24] with agar plugs or conidia from freshly sporulated PDAB plates. Germination on glass surfaces was tested as described by Doehlemann et al. [11]. Penetration ability was checked on onion epidermal layers. Conidia were washed and placed on epidermal layers of onions. Before microscopy, the hyphae growing on top of the layers were stained with lactophenol blue. For observation of infection cushions (ICs) the epidermal layers were incubated with agar plugs overnight [44]. For visualization of conidial anastomosis tubes (CATs) media and strains were prepared following [44].

### Quantification of conidia

For quantification of conidia a defined amount of spores (0.5 × 10^5^) were plated on CM plates. After 14–21 days of incubation under daytime rhythm, the spores were washed down with 10 µl H2O, followed by a second wash step (5 ml) to harvest the remaining spores. Dilutions were prepared and spores were quantified in several replicates using a Thoma chamber.

### ROS quantification

Quantification of ROS levels was accomplished by applying the Total ROS/superoxide detection kit (Enzo Lifescience, Lausen, Switzerland). The detection mix was prepared as described in the instructor’s manual. For visualization, conidia were harvested from a freshly grown PDAB plate and 100 µl (10^5^ conidia/ml) were grown overnight in a 96-well plate (Microplate pureGradeTM 96 well PS, transp. Bottom, black, Brand GmbH & Co KG, Germany). Just before measurement the detection mix was added (1/10 vol). Pictures were taken using the wavelengths 490/525 (Ex/Em). For every well 3 × 3 reads were done with a gain that was set to 200.

### Epifluorescence microscopy

Light microscopy imaging was performed using the Axio Imager 2 and the Axiovert (Zeiss, Jena, Germany). Visualization of infection cushions was done with the 20× objective lens, while germinated conidia were analyzed with 40× or 63× magnification. ER staining was accomplished using the ER-Tracker™ Blue-White DPX (Life Technologies, Germany) in McIlvaine standard buffer [35]. The samples were observed via the filter set 49 DAPI shift free (excitation G 365, beam splitter FT 395, emission BP 445/50). GFP fluorescence was detected with filter set 38 (excitation BP 470/40, beam splitter FT 495, emission BP 525/50). Images were captured with a Zeiss AxioCamMRm camera and further processed using the AxiovisionRel 4.8 software package.

### Confocal laser scanning microscopy (CLSM) imaging and ratiometric analysis

roGFP2 measurements were done using an inverted microscope (Leica DMIRE2) equipped with a Leica TCS SP2 scan head (Leica Microsystems, Wetzlar, Germany). Conidia were prepared as described before [20]. Results were obtained by using the excitation wavelengths 395 (first track) and 488 (second track) as well as a 505–530 bandpass filter for collecting images. Z-stacks were displayed as average projections via the CLSM software. Further evaluation was done with the Image J program (v.1.44f; http://rsb.info.nih.gov/ij/) as it was shown before [33].

For measuring ROS levels, a Tecan Safire reader was used. Conidia were harvested and placed into a 96-well plate Microplate pureGradeTM 96 well PS, transp. Bottom, black, Brand GmbH & Co KG, Germany). After growth overnight (10^5^ conidia/ml) the detection mix was added and fluorescence was measured with 3 × 3 reads/well and excitation/emission as described above. The gain was set to 200.

### Database resources

Nucleotide and protein sequences of *B.* *cinerea* strain B05.10 were obtained by the database Ensembl (http://fungi.ensembl.org/Botrytis_cinerea/Info/Index). For the analysis of the sequences, different programs were used: Signal peptides were predicted by SOSUIsignal [17], subcellular localization patterns of proteins were predicted by ProtComp v.9.0 (http://linux1.softberry.com/berry.phtml?topic=protcompan&group=help&subgroup=proloc), transmembrane regions were predicted by using the programs TMHMM (http://www.cbs.dtu.dk/services/TMHMM/) and SACS MEMSAT2 [22] as well as PRED-TMR [36].

## Results

In *B.* *cinerea*, two Nox complexes are responsible for important vegetative and pathogenic growth and differentiation processes [43, 44]. The most recent studies focused on the identification of new members of both Nox complexes [32, 43, 44]. However, information about Nox complex regulation, site of action and direct linkages to existing signaling pathways remained scarce.

### Transcriptional regulation of BcNoxA/B is not important for their distinct function

To elucidate whether transcriptional regulation of both catalytic subunits does have any influence on their function, hybrid constructs were generated. The coding region of *bcnoxA* was cloned downstream of the *bcnoxB* promoter with (C1) or without (C2) the N-terminal elongation of *bcnoxB*, while the coding region of *bcnoxB* was fused to the promoter region of *bcnoxA* (C3, Fig. 1).

All constructs were transformed in the deletion mutants of *bcnoxA* or *bcnoxB,* respectively and integrated into the *bcniaD* or *bcniiA* locus (see “Materials and methods”, Additional file 1: Table S1 as well as [39]. Since BcNoxA is involved in formation and fusion of conidial anastomosis tubes (CATs—specialized hyphae that facilitate the exchange of cellular material), infection cushions (IC—bulbous aggregates that facilitate the penetration of plant tissue) and sclerotia as well as affects the colonization of plant tissue, the Δ*bcnoxA* transformants were investigated with special focus on these phenotypes. Only the construct C2 was able to fully complement the phenotype of Δ*bcnoxA* (Fig. 1a–g) whereas the fusion protein C1 only partially restored the wild type phenotype. Thus, mutants containing the C1 construct displayed wild type like pathogenicity (Fig. 1a, b) and a restored formation of infection cushions (Fig. 1g), but produced a minor amount of CATs (Fig. 1c), sclerotia (Fig. 1d) and conidiospores (Fig. 1e). Mutants with an integrated C3 construct displayed the deletion mutants’ phenotype.

Since BcNoxB affects the production of functional appressoria, the penetration of plant tissue, sensitivity against H_2_O_2_ as well as the production of ROS and conidiospores, all transformants (Δ*bcnoxB* background) were analyzed with special focus on these phenotypes.

While mutants containing the constructs C1 or C2 resembled the phenotype of Δ*bcnoxB*, mutants expressing the C3 construct appeared to be wild type like (Additional file 4: Figure S4). Thus, the construct C3 mediates the restoration of full pathogenicity (Additional file 4: Figure S4A), the formation of functional appressoria (Additional file 4: Figure S4B), conidiation (Additional file 4: Figure S4C) as well as stress resistance (Additional file 4: Figure S4D) and ROS production (Additional file 4: Figure S4E).

In conclusion, the catalytic subunits of the Nox complexes fail to replace each other, despite of their structural similarities. Moreover, the N-terminal elongation (Additional file 3: Figure S3B) of BcNoxB seems to compromise the folding of BcNoxA since the construct C1 is not sufficient to fully restore the phenotype of Δ*bcnoxA*. Finally, unexpectedly the transcriptional regulation (exchange of promoters) appears to be not important for the distinct functions of Nox complexes.

### Membrane orientation of BcNoxA would allow electron transport into the ER

**Fig. 2:**
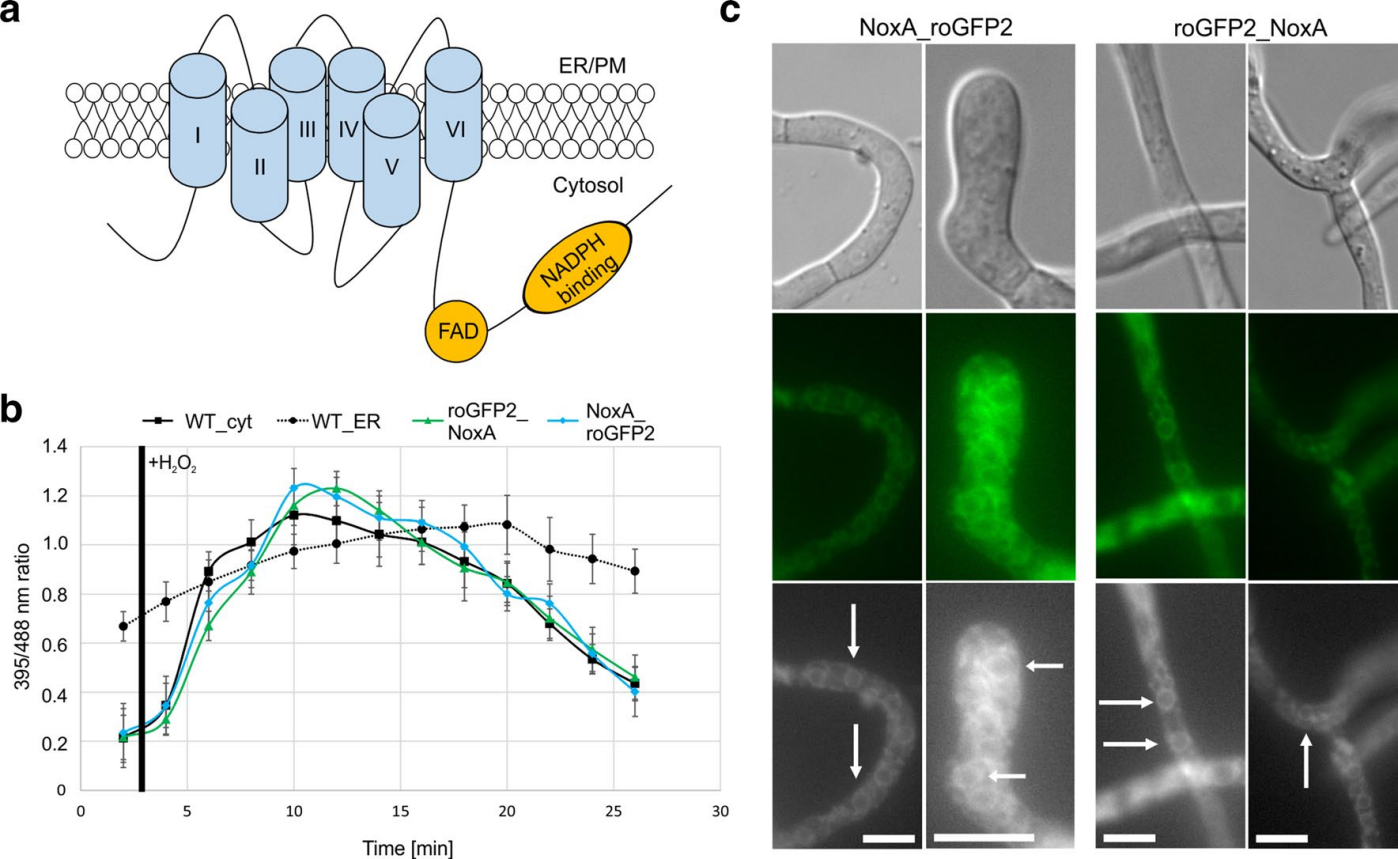
RoGFP2 fused to BcNoxA reports cytosolic redox state changes. The genetically encoded biosensor roGFP2 was fused to the N- and C-terminus of *bcnoxA*. **a** NoxA is predicted as protein containing six transmembrane domains with both ends located inside the cytosol. Prediction was accomplished by the program PRED-TMR [36]. **b** For roGFP2 measurements, conidia were incubated on microscopic CLSM slides overnight in B5 medium supplemented with glucose (2 %). Measurements were performed by using the excitation wavelengths 395 (first track) and 488 (second track) as well as a 505–530 bandpass filter for collecting images. The ratio of 395/488 images was build and compared to the reference strains (B05.10 with cytosolic or endoplasmic roGFP2). For the induction, the medium was removed and replaced by B5 medium supplemented with glucose (2 %) and 10 mM H_2_O_2_. **c** Microscopic analyses of the roGFP fusion constructs revealed that the protein (as described before) is localized to ER structures. For the microscopic analyses the strains were grown in B5 medium supplemented with 2 % glucose overnight on glass slides. *White arrows* are indicating perinuclear structures, which belong to the ER. *Scale bars* 10 µm

BcNoxA is predicted to be a transmembrane protein with N- and C-terminus located inside the cytosol (Fig. 2a). Thus, the protein would be able to bind cytosolic NADPH at its C-terminal NADPH-binding-domain and transfer the electrons across biological membranes onto molecular oxygen. Since it was shown that the genetically encoded biosensor roGFP2 is suitable for topology experiments [50], the gene encoding the reporter protein was fused to the N- and C-terminus of *bcnoxA*. The biosensor roGFP2 specifically senses the redox potential of the intracellular glutathione pool and appears to be nearly unaffected by pH changes [41]. Depending on the redox state (in more detail: the ratio of oxidized and reduced glutathione), the conformation of the roGFP2 protein changes, leading to different spectral properties measured at 395 and 488 nm. In previous studies, the biosensor was integrated into the cytosol [20], into the intermembrane space of mitochondria and the ER [33]. While the cytosol displayed reduced conditions (_395/488_ratio = 0.2), the ER was the most oxidized compartment (_395/488_ratio = 0.7). In both compartments, the _395/488_ratio changes upon exposure to H_2_O_2_ (Fig. 2b). Dependent on the localization of the N- and C-terminus of BcNoxA, roGFP2 reflects the redox state either of the cytosol or the one of the ER.

After generating the strains B05.10_roGFP2_NoxA and B05.10_NoxA_roGFP2, the correct integration of the fusion constructs (see “Materials and methods”, Additional file 1: Table S1) was checked via diagnostic PCR. The functionality of both constructs was verified before, by complementing Δ*bcnoxA*. Both proteins (roGFP2_NoxA/NoxA_roGFP2) appeared to be localized to the plasma membrane, perinuclear regions as well as filamentous ER structures (Fig. 2c); confirming the localization pattern observed for BcNoxA_GFP [44]. Fluorometric measurements with both roGFP2 versions (located at the N- or C-terminus of BcNoxA) resulted in the report of a typically cytoplasmic redox state (Fig. 2b), suggesting that both protein ends are located inside the cytosol. In both cases the untreated _395/488_ratio (wild type: 0.22) displays values of 0.23 (NoxA_roGFP2) and 0.21 (roGFP2_NoxA). Upon exposure to H_2_O_2_, the _395/488_ratio increases up to its maximum after 8 min post induction in the cytosolic wild type control as well as in the strains NoxA_roGFP2 and roGFP2_NoxA. A similar behavior upon H_2_O_2_ induction was observed in previous studies [20, 33].

These results suggest that the membrane orientation of BcNoxA would allow the transport of electrons from the cytosol across the ER- or plasma membrane. In the end, this ROS production inside of the ER as well as into the extracellular space would be possible.

### The N-terminus of BcNoxA is essential for its ER functions

Since it was shown that an ER-hooked allele of BcNoxA is only partially able to complement the deletion mutant’s phenotype [44], we analyzed the importance of the predicted N-terminal signal peptide of BcNoxA. For this, an allele, which encodes for a truncated version of BcNoxA (loss of the first 41 aa—Additional file 3: Figure S3A) was transformed into the Δ*bcnoxA* mutant.

**Fig. 3:**
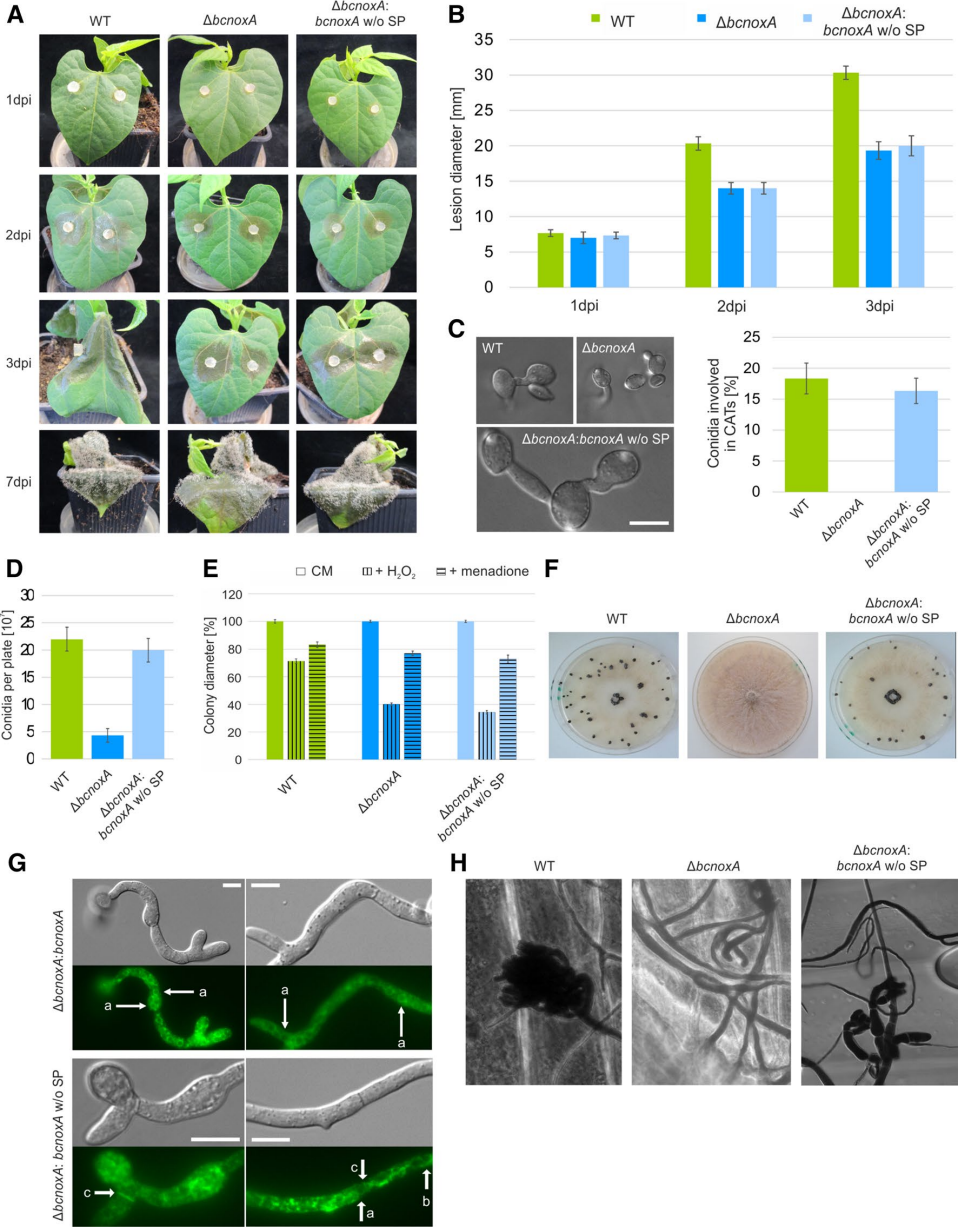
The N-terminus of BcNoxA is essential for full functionality. The deletion mutant of *bcnoxA* was complemented by *bcnoxA* or a N-terminal truncated version of *bcnoxA*. The construct was integrated into the *bcniiA* locus (Additional file 1: Table S1). **A**, **B** The truncated *bcnoxA* version is not able to infect bean leaves as the wild type. Bean leaves were inoculated with agar plugs. Lesion diameters were measured and statistically evaluated. Replicates showed similar results. **C** Δ*bcnoxA*:*bcnoxA* w/o SP is able to produce hyphal fusions. Conidia were incubated on minimal medium for 18 h. Hyphal fusions were detected microscopically and evaluated statistically (300 spores each). **D** Δ*bcnoxA*:*bcnoxA* produces wild type like amounts of conidiospores. The strains were incubated on CM medium for at least 3 weeks. Conidia were harvested, diluted and counted. Replicates showed similar results. **E** Tests for stress sensitivity reveal that Δ*bcnoxA*:*bcnoxA* w/o SP is equally sensitive to oxidative stress such as H_2_O_2_ (10 mM) or menadione (500 µM) as the deletion mutant of *bcnoxA*. Agar plugs were set on CM and selective media and monitored for seven days (here depicted: 3 dpi). **F** Sclerotia production is restored in the strain Δ*bcnoxA*:*bcnoxA* w/o SP. Agar plugs were incubated on CM in constant darkness for at least 14 days. **G** BcNoxA w/o SP-GFP has a different localization pattern compared to BcNoxA-GFP. Conidia were incubated overnight on microscopic slides and analyzed microscopically. The untruncated version can be found in the nuclear envelop (*a*), while the truncated version is located in filamentous structures (*b*) as well as in the region of septa (*c*). **H** Δ*bcnoxA*:*bcnoxA* w/o SP is still impaired in the production of infection cushions. Onion epidermal layers were incubated with agar plugs for at least 20 h. Just before microscopy the fungal material on the surface of the epidermal layers was stained with lactophenol blue. *Scale bars* 10 µm

Phenotypic analyses revealed that the protein is not able to restore full pathogenicity (Fig. 3A, B) as well as the formation of infection cushions (Fig. 3H). Likewise, the mutant strain is still more sensitive to oxidative stress (Fig. 3E). In contrast, phenotypes such as the formation of CATs (Fig. 3C), conidiospores (Fig. 3D) and sclerotia (Fig. 3F) were complemented by the truncated version of BcNoxA. Surprisingly, these results are inverse to those obtained with the ER-hooked version of BcNoxA [44].

Microscopic analyses show that the localization pattern changes in comparison to the non-truncated version. While BcNoxA_GFP localizes to filamentous ER structures and the nuclear envelope (Fig. 3G (a)) as reported [44], the truncated BcNoxA_GFP is found in a faint network of filamentous structures (Fig. 3G (b)), septa (Fig. 3G (c)) and was only rarely observed in the nuclear envelope (Fig. 3G (a)). Summarized, the N-terminus of BcNoxA seems to be essential for the proper localization of the protein as well as for its functions inside of the ER.

### ER-hooked alleles of BcNoxD and BcNoxA are involved in identical processes

BcNoxD, a putative ER membrane protein was shown to be a new member of the NoxA complex in *B.* *cinerea* [44]. Deletion mutants of *bcnoxD* resemble the phenotype of Δ*bcnoxA* and both proteins directly interact in yeast-2-hybrid and co-IP assays. To investigate whether BcNoxD has also distinct functions inside and outside of the ER as it was previously confirmed for BcNoxA, an ER retention signal was fused to the 3′ end of the *bcnoxD* coding region (BcNoxD_HDEL).

The ER-hooked allele of BcNoxD is able to restore the formation of IC’s and the colonization defect of its deletion mutant (Additional file 5: Figure S5A/B/G). Moreover, BcNoxD_HDEL restores the resistance against oxidative stress (Additional file 5: Figure S5F) which was also demonstrated for the ER-hooked allele of BcNoxA (Additional file 6: Figure S6). In contrast, the mutant strain is still impaired in the formation of CATs, sclerotia and conidiospores (Additional file 5: Figure S5C/D/E). Summarized, both strains Δ*bcnoxA*:*bcnoxA*_HDEL and Δ*bcnoxD*:*bcnoxD*_HDEL display an identic functional pattern, i.e. the native proteins have identical distinct functions within and outside the ER.

### BcNoxB has a role inside and outside the ER

Since previous studies suggested a distinct function of the NoxA complex inside the ER, further studies were conducted to elucidate the role of the NoxB complex inside and outside the ER. As previously done for BcNoxA and BcNoxD, the ER retention signal “HDEL” was fused to the 3′ end of the *bcnoxB* coding region. The construct was transformed into Δ*bcnoxB* and integrated into the *bcniaD* locus [39].

**Fig. 4:**
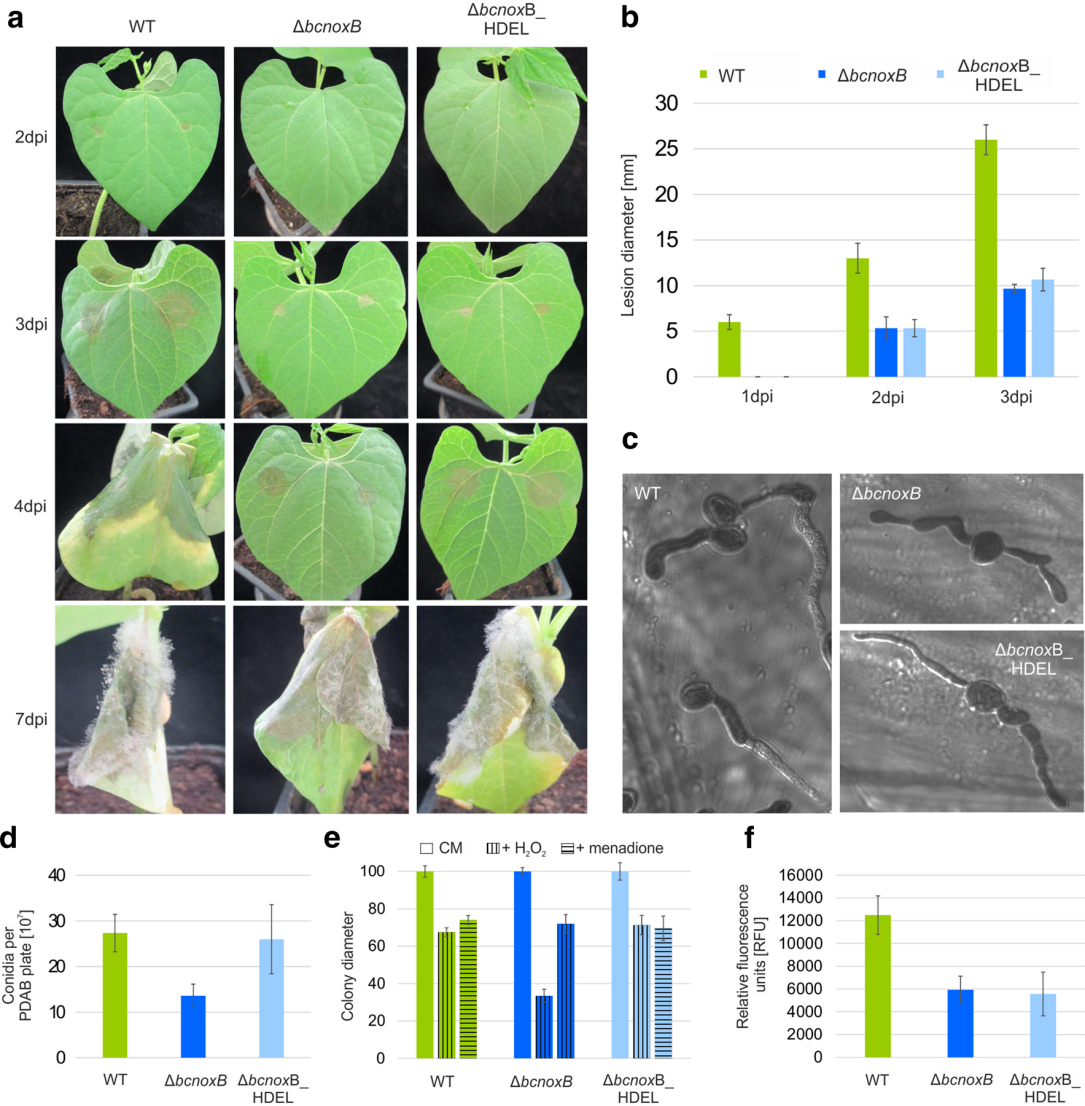
BcNoxB has its predominant role outside the ER. Δ*bcnoxB* was complemented with an ER locked allele of *bcnoxB*. The construct was integrated into the *bcniaD* locus (Additional file 1: Table S1). **a**, **b** The ER retained version of BcNoxB is not able to infect bean leaves as good as the wild type. Primary bean leaves were inoculated with 7.5 µl of conidial suspension (10^5^ conidia/ml). Lesion diameters were measured and statistically evaluated (3 bean plants/strain). Replicates showed similar results. **c** Δ*bcnoxB* and Δ*bcnoxB*:*bcnoxB*_HDEL are not able to form functional appressoria for the penetration of onion epidermal layers. Onion epidermal layers were infected with drops of a conidial suspension (10^5^ conidia/ml). Just before microscopy the fungal hyphae were stained with lactophenol blue. **d** The ER locked allele of BcNoxB is able to produced wild type like levels of conidiospores. The different strains were incubated on CM medium for at least 3 weeks. Conidia were harvested, diluted and quantified. Replicates showed similar results. **e** Stress resistance is mediated by the ER retained version of BcNoxB. Agar plugs were placed on CM agar and selective media supplemented with H_2_O_2_ (10 mM) or menadione (500 µM) and monitored for 7 days (here depicted: 3 dpi). **f** BcNoxB_HDEL is not sufficient for restoration of wild type like ROS levels. Spores were grown in a microtiter plate overnight. Just before microscopy the detection agent for the visualization of ROS was added. Monitoring took place in a Tecan Saphire with 3 × 3 reads. Replicates displayed similar results

In contrast to the ER locked allele of BcNoxA, the modified version of BcNoxB is not able to restore full pathogenicity, when hooked to the ER membrane (Fig. 4a, b). Moreover, Δ*bcnoxB*:*bcnoxB*_HDEL produced no functional appressoria (Fig. 4c) and displayed reduced levels of ROS (Fig. 4f) comparable to Δ*bcnoxB*. Nevertheless, the ER does not appear to be just a storage compartment for the second catalytic subunit BcNoxB, since important processes such as the production of conidiospores and resistance against H_2_O_2_ seem to be affected by the ER locked allele of BcNoxB (Fig. 4d, e).

In conclusion, the NoxB complex of *B.* *cinerea* seems to have a role inside and outside the ER, most likely at the plasma membrane. While stress sensitivity and conidiospore production are regulated by BcNoxB inside of the ER, the protein affects ROS production and pathogenicity outside the ER.

## Discussion

The production of ROS and the cellular redox homeostasis are essential for the plant pathogen *B. cinerea* and its interaction with the host. By producing superoxide, Nox complexes directly contribute to elevated levels of ROS and directly influence the redox balance of the fungus [33]. Since the link between Nox-function and differentiation processes of filamentous fungi were identified [28], many studies aim to uncover Nox-function, composition, regulation and the integration in the complex signaling network.

**Fig. 5:**
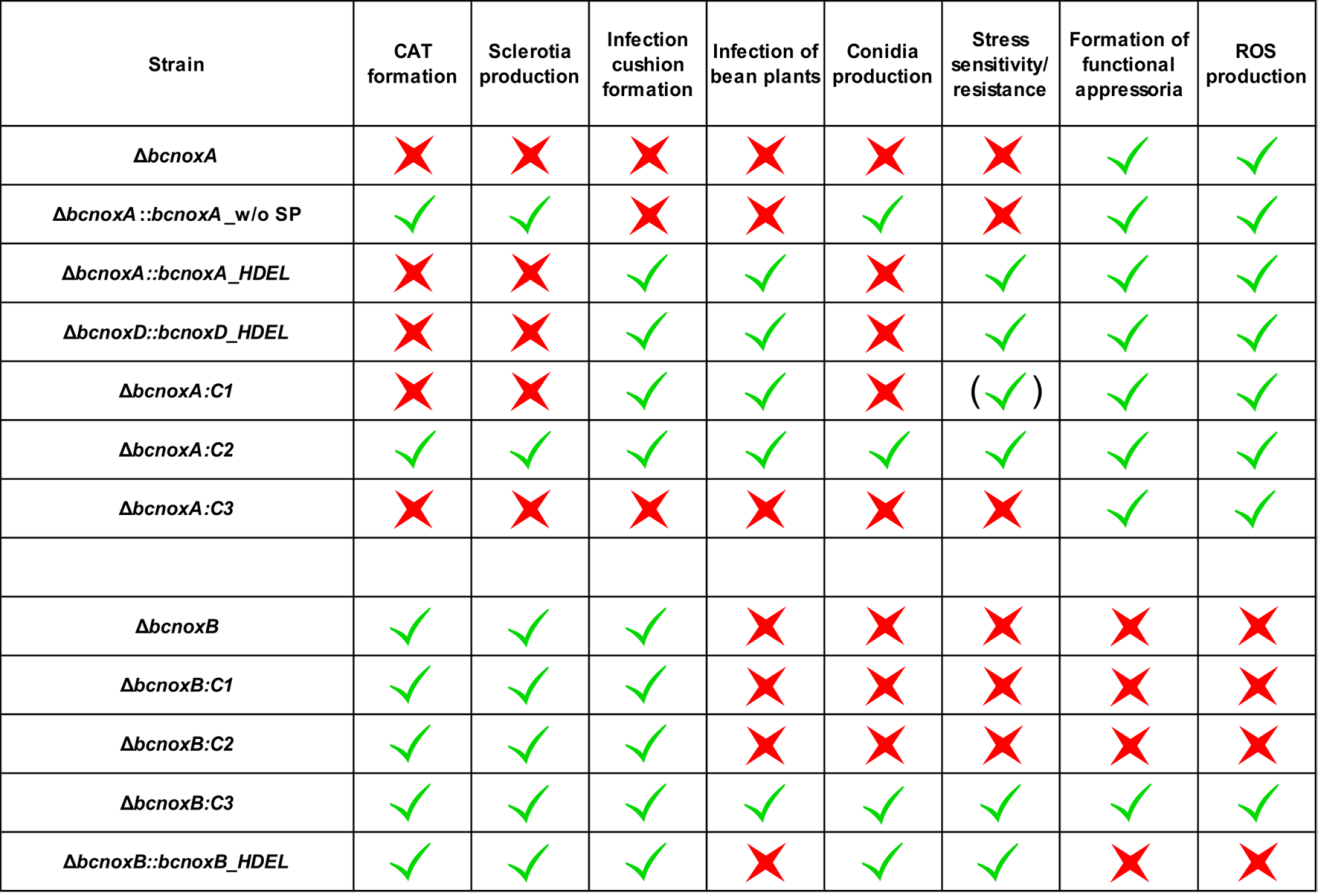
Nox complexes and their composition dependent on their site of action and function. Overview of the mutants’ phenotype in comparison to the wild type and the growth and differentiation pattern of Δ*bcnoxA* and Δ*bcnoxB*. *Red crosses* = mutants have defects in the respective differentiation process; *green ticks* = mutants display a wild type like phenotype

In previous studies, we were able to illustrate that in *B. cinerea* there are two Nox complexes with—for the most part—distinct functions [43, 44]. Both proteins are located at the ER and plasma membrane and at least for the NoxA complex specific functions inside and outside the ER were suggested. Thus, an ER hooked allele of BcNoxA was shown to complement pathogenicity and the formation of infection cushions (Fig. 5) [44].

In this study, we elucidate the orientation of BcNoxA in the lipid bilayer by the genetically encoded biosensor roGFP2. The biosensor, which was fused to the N- and C-terminus end of *bcnoxA*, can be used as a tool to study membrane topologies and for answering the question, whether the orientation of the catalytic subunit would allow ROS production e.g. inside the ER. CLSM analyses reveal that both ends of the protein are located inside the cytosol (Fig. 2). Thus, the biosensor mirrored a greatly diminished _395/488_ratio (indicating a compartment with reduced conditions) and, therefore, resembled results of previous studies that proposed the cytosol of *B.* *cinerea* as compartment with the most reduced conditions [20, 33]. Since the NADPH binding domain is predicted to be at the very end of the C-terminus of BcNoxA, the orientation would allow the transfer of electrons from the cytosol across membranes e.g. into the ER lumen (Fig. 2a). The activity of the NoxA complex would lead to an oxidation of the cytosol and the reduction of molecular oxygen on the other site of the lipid bilayer. A similar mechanism was observed previously in the mammalian system, where the dual oxidase 2 (Duox2—belonging to the Nox family) produces superoxide inside the ER in a calcium dependent manner [2]. Moreover, this suggested pattern was recently confirmed by ratiometric measurements using the genetically encoded biosensor roGFP2 in the wild type and deletion mutants of *bcnoxA* in *B. cinerea* [33].

For further investigations of the coherence between Nox structure and function, we modified the N-terminus of BcNoxA by removing the putative signal peptide (Fig. 3/Additional file 3: Figure S3A). In mammals, a direct link between N-terminus and localization as well as type of ROS was already illustrated [21]. In the fungal system, the truncation of the N-terminal region affected both, localization and function of BcNoxA. In contrast to the wild type version of BcNoxA, the protein was rarely observed in the nuclear envelope, but seemingly more often in filamentous structures of the ER (Fig. 3G (b)). Complementation studies revealed that the truncated version of BcNoxA is able to complement the deletion mutants’ phenotype only partially. Thus, the formation of sclerotia and CATs was restored (Fig. 3C, F), whereas IC formation and pathogenicity were still disturbed (Fig. 3A, B, H). Interestingly, the complemented phenotypes were inverse to the ones of the ER hooked allele (Fig. 5) [44]. However it remains elusive, whether the inverse phenotype is due to the diverging localization of the protein or due the missing interaction to other Nox complex members, both attributable to the missing signal peptide. Nevertheless, these data substantiate that the the NoxA complex has distinct functions in different cellular compartments and suggest that the N-terminal region of BcNoxA is essential for its function inside the ER. This hypothesis is further supported by cross complementation studies, which aimed to uncover the importance of the transcriptional regulation of *bcnoxA/bcnoxB* for their protein function. After complementing Δ*bcnoxA* with the respective constructs (C1–C3, Fig. 1) our data indicate that only *bcnoxA* under the control of P_*bcnoxB*_ (C2) is sufficient to complement all phenotypes of the deletion mutant. Surprisingly, the N-terminal elongation of *bcnoxB* in construct C1 prevents full complementation of the respective deletion mutant. The complemented strain resembled the phenotype of Δ*bcnoxA::bcnoxA*_HDEL. This underlines the importance of the N-terminal region for the proper localization of BcNoxA and coincides with the previous reported results in mammalian systems [21].

Even more captivating are the cross complementation studies after considering the results of the respective Δ*bcnoxB* complementation. Our final data reveal that only the construct containing the full-length version of *bcnoxB* is sufficient to restore the wild type phenotype (Additional files 4: Figure S4, 5: Figure S5). In conclusion, the results strongly suggest that (1) the catalytic subunits are not able to replace each other, (2) the N-terminal elongation of BcNoxB is not able to transfer characteristics of BcNoxB onto BcNoxA and most important (3) the transcriptional regulation is not important for BcNoxA/B function since the successful cross complementation was promoter independent. Especially the irrelevance of the transcriptional regulation for pathogenic and vegetative differentiation processes is striking. Thus, previous studies in mammals revealed that the expression pattern of the different Nox isoforms is essential for their function and that distinct signaling pathways regulate the expression of the different isoforms (reviewed in [26, 42]). Nevertheless, in *B.* *cinerea*, post-translational modifications and mechanisms that directly affect the respective proteins may be more important than the transcriptional regulation. Thus, (calcium-dependent) phosphorylation events, glycosylation, nitrosylation or direct redox dependent regulation of cysteine residues might affect Nox complex activity as seen in plant and mammalian cells (summarized in [30]; reviewed in [16, 46, 47].

**Fig. 6:**
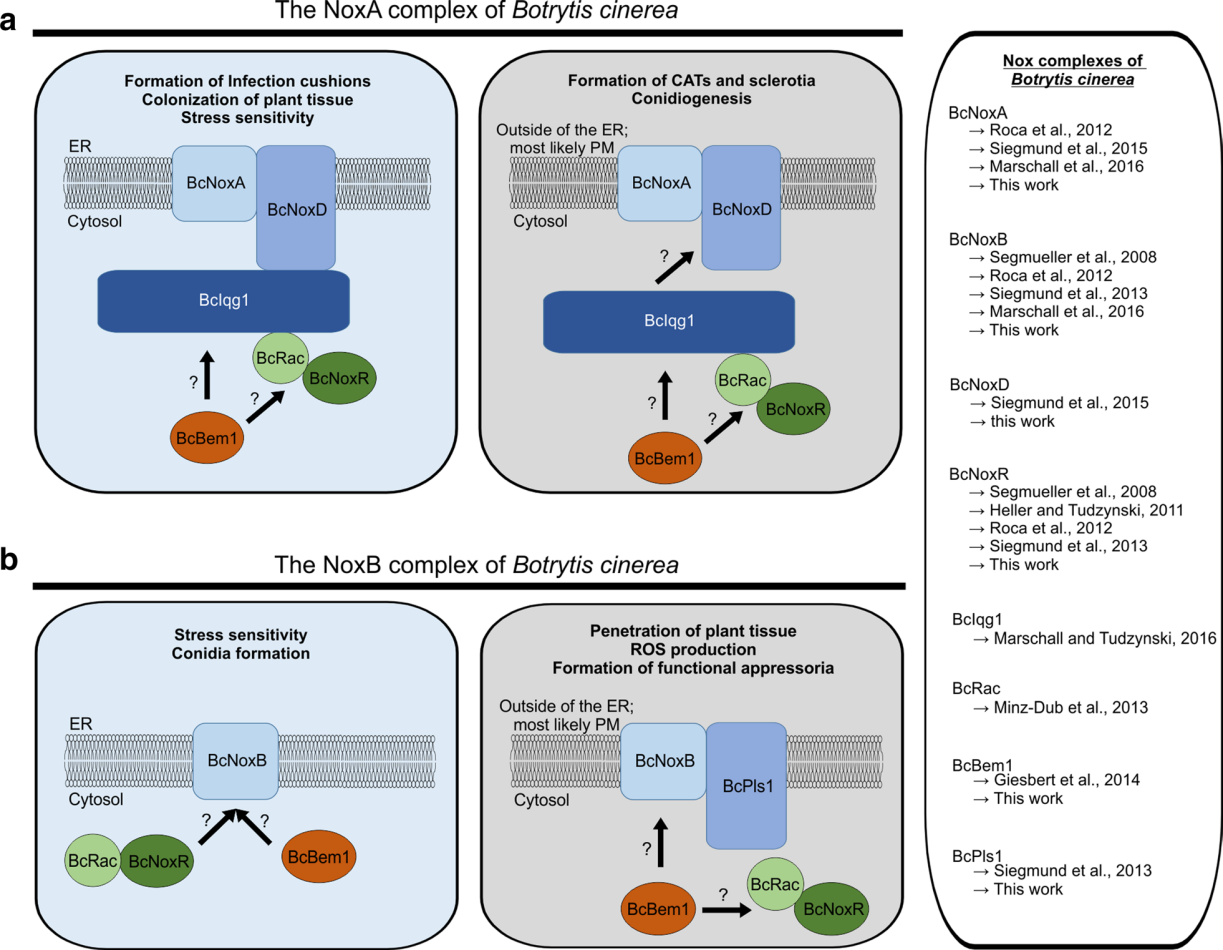
Hypothetical model for the compositions of the NoxA (**a**) and NoxB (**b**) complexes during distinct differentiation processes. For detailed description see text. Publications, which describe the respective phenotypes are listed right beside it

Despite of its own regulation, the catalytic subunits require further subunits (e.g. BcBem1 and Rac) to build up a functional complex. Depending on the regulated process and the site of action, several subunits putatively contribute to the assembly and full functionality of both Nox complexes (Fig. 6). As reported previously, the NoxA complex of *B.* *cinerea* especially requires its adaptor protein BcNoxD and the putative regulator BcNoxR, which exhibit identical or at least overlapping phenotypes [42–44]. In this work, we confirmed the substantial role of BcNoxD for the NoxA complex by unravelling its role inside the ER. Thus, we were able to show that an ER locked allele of BcNoxD complements the identical phenotypes as for the previously analyzed BcNoxA allele (Additional file 5: Figure S5) [44]. In all experiments with an ER locked allele, it is noteworthy that despite the ER retention signal there might also be a small amount of proteins present outside the ER.

By considering all phenotypes of putative Nox complex members, we propose a model of NoxA complex compositions dependent on the site of action and the respective differentiation process (Fig. 6a). Thus, the NoxA complex might regulate pathogenic processes such as colonization of plant tissue and infection cushion formation as well as vegetative processes such as stress resistance inside the ER (Additional file 6: Figure S6A/Fig. 6). In this situation, the NoxA complex consists of the membrane standing subunits BcNoxA and BcNoxD, which are putatively linked via the scaffold protein BcIqg1 to its cytosolic and regulatory subunits BcRac as well as BcNoxR. Since the deletion mutant of the second scaffold protein BcBem1 displays also defects in the colonization of plant tissue [15] and in the formation of infection cushions (Additional file 6: Figure S6B) an association to the NoxA complex is likely. The putative association is supported by Takemoto et al. (2011), who linked Bem1 to Nox complexes in *Epichloe festucae* [48]. On the other side, the unaffected stress resistance of Δ*bcbem1*, its lacking Nox complex interaction partner in *B.* *cinerea* as well as its localization in the cytosol and septa [15] cast some doubts about its membership in the NoxA complex. Outside the ER, the NoxA complex might be similar composed during the formation of CATs and conidiospores, but slightly different during sclerotia development. Δ*bcbem1* and Δ*bciqg1* were shown to produce higher amounts of sclerotia than the wild type [15, 32]. This might hint to a negative regulation mechanism either, or to a different composition of the NoxA complex with respect to this differentiation process.

To unravel the site of action and regulation processes of the NoxB complex in *B.* *cinerea*, a similar complementation approach with an ER locked allele was conducted. In contrast to BcNoxA, the second catalytic subunits has its main functions outside the ER. Thus, the formation of functional appressoria and the related penetration of plant tissue as well as the production of ROS were not complemented by Δ*bcnoxB::bcnoxB*_HDEL (Figs. 4, 5). Since previous studies already indicated that the second catalytic subunit has a predominant role in the ROS production [33], it is reasonable that those massive amounts most likely are generated at the plasma membrane towards the extracellular space. Here, ROS may act against the host defense barrier or may be transported back inside the fungal hyphae via evolved transport systems, such as aquaporins [3]. The latter hypothesis is supported by previous findings that superoxide accumulates inside the hyphal tip during the formation of penetration structures [34]. A direct production e.g. inside the ER/cytosol may lead to an intoxication and cell death.

Strikingly, only the stress resistance is regulated by both Nox complexes inside the ER, suggesting that both complexes contribute to the sensing machinery of oxidative (Fig. 4; Additional file 5: Figure S5A), osmotic and cell wall stress (data not shown) when hooked to the ER membrane. In contrast, BcNoxB inside the ER affects conidiation, while NoxA contributes to this differentiation process outside the ER. A similar interplay between both Nox complexes was recently observed upon calcium stress exposure [33].

As previously discussed for the NoxA complex, also the composition of the NoxB complex slightly differs dependent on the site of action as well as on the differentiation process (Fig. 6b). Inside the ER, the catalytic subunit seems to work as single membrane standing protein since its putative adaptor protein BcPls1 neither affects conidiation nor contributes to stress resistance of *B.* *cinerea* [43]. In contrast, all other processes, which are putatively regulated by BcNoxB outside the ER, might be affected by a complex consisting of the catalytic subunit (BcNoxB), the tetraspanine BcPls1, the regulator BcNoxR as well as the cytosolic members BcRac and BcBem1 (Fig. 6b). However, the link between BcPls1 and BcNoxB appears to be weak and might be restricted to specific time points and differentiation processes, which is also supported by the lacking physical interaction of both proteins in yeast based interaction assays as well as in co-IP and pulldown experiments (data not shown). The contribution of BcBem1 to the development of functional appressoria needs to be further elucidated, since Δ*bcbem1* is able to form functional penetration structures [15]. However, all subunits display penetration defects on bean plants as well as a reduced amount of secreted ROS (Additional file 6: Figure S6C). Processes such as sclerotia formation are not regulated by the NoxB complex (Additional file 6: Figure S6D).

## Conclusion

In conclusion, we can use our obtained data to propose a putative model for both Nox complexes dependent on their site of action and their function. Strikingly, both complexes have distinct functions inside and outside the ER and as previously seen in the mammalian system, the compositions might differ dependent on the respective differentiation process. According to bioinformatic analyses, we confirmed that the orientation of the catalytic subunit BcNoxA would allow the production of ROS inside the ER lumen. Remarkable is, that against previous observations and assumptions, the transcriptional regulation of both Nox complexes seems to be unimportant for their distinct functions. Nevertheless, studies are needed to further elucidate the integration of both complexes in the up- and downstream wiring as well as the exact composition and connections of the catalytic subunits with its regulator as soon as the respective scaffold and adaptor proteins are missing.

## Additional files

**Additional file 1: Table S1.** Strains used in this work.

**Additional file 2: Table S2.** Oligonucleotides used in this work.

**Additional file 3: Figure S3.** Bioinformatic analysis of BcNoxA and BcNoxB. (A) Protein sequence of BcNoxA with the highlighted putative signal peptide. The prediction was accomplished using the SOSUIsignal algorithm. (B) Alignment of BcNoxA and BcNoxB using the Clustal Ω algorithm. The N-terminal elongation of NoxB was used in the cross complementation experiments. Asterisk highlight identical amino acids, while colons are indicating amino acids with similar characteristics. Dots refer to a faint resemblance.

**Additional file 4: Figure S4.** Transcriptional regulation is not important for NoxB function. Three complementation constructs (C1–C3) were generated and transformed in Δ*bcnoxB*. Integration took place into the *bcniiA* locus (Table S1). (A) Wild type like spore-mediated infection is restored in Δ*bcnoxB*:C3. Primary bean leaves were infected with 7.5 µl of conidial suspension (10^5^ conidia/ml). Lesion diameters were measured and statistically evaluated (3 bean plants/strain). (B) Δ*bcnoxB,* Δ*bcnoxB*:C1 and Δ*bcnoxB*:C2 are deficient in forming functional appressoria. Onion epidermal layers were infected with drops of a conidial suspension (10^5^ conidia/ml). Just before microscopy the fungal hyphae were stained with lactophenol blue. (C) Only Δ*bcnoxB*:C3 produces the same amount of conidiospores as the wild type. Spores were harvested from CM plates with 10 ml H_2_O and quantified in replicates. (D) Plate assays for the determination of the stress resistance display identical results for the wild type and the strain Δ*bcnoxB*:C3. Agar plugs were placed on CM agar and medium supplemented with H_2_O_2_ (10 mM) or menadione (500 µM). Monitoring was accomplished for seven days. (E) Only Δ*bcnoxB*:C3 produces wild type like levels of ROS in microplate assays with the TRD kit. Spores were grown in a microtiter plate for 12-16 h. Just before microscopy the detection agent for the visualization of ROS was added. Monitoring took place in a Tecan Saphire with 3 × 3 reads. Replicates displayed similar results. Scale bars = 10 µm.

**Additional file 5: Figure S5.** BcNoxD_HDEL resembles the phenotype of BcNoxA_HDEL and has functions inside and outside the ER. Δ*bcnoxD* was complemented with an ER locked allele of *bcnoxD*. The construct was integrated into the *bcniaD* locus (Table S1). (A/B) The infection defect of Δ*bcnoxD* is fully restored by the ER locked allele of BcNoxD. Bean leaves were inoculated with conidiospores (10^5^ conidia/ml) and monitored until the tissue was fully macerated. Lesion diameters were measured and statistically evaluated (3 bean plants/strain). (C) The ER bound allele of NoxD is not sufficient to restore the formation of hyphal fusions. Conidia were incubated on minimal medium for 18 h. Hyphal fusions were detected microscopically and evaluated statistically (300 spores each). (D) Sclerotia production is restored in the strain Δ*bcnoxA*:*bcnoxD_*HDEL. Agar plugs were incubated on CM in constant darkness for at least 14. (E) Δ*bcnoxA*:*bcnoxD_*HDEL displayed reduced levels of conidiospores. Spores were washed down from CM plates with 10 ml H_2_O and quantified in three replicates. (F) Stress resistance was tested on CM agar in comparison to selective media supplemented with H_2_O_2_ (10 mM) or menadione (500 µM) for seven days. Colony diameters were measured (here depicted: 3 dpi). The ER locked allele mediates resistance against oxidative stress conditions. (G) Infection cushion formation is restored in Δ*bcnoxA*:*bcnoxD_*HDEL. Onion epidermal layers were inoculated with agar plugs of the respective strains. Staining of fungal hyphae was performed with lactophenol blue just before microscopy.

**Additional file 6: Figure S6.** Differentiation processes affected by members of Nox complexes in *B. cinerea* (A) BcNoxA mediates stress resistance when hooked to the ER membrane. Δ*bcnoxA* was complemented with an ER locked allele of *bcnoxA* and tested for stress sensitivity against oxidative stress agents such as H_2_O_2_ (10 mM) or menadione (500 µM). Stress resistance is fully restored by the ER locked allele of BcNoxA. Colony diameters were measured for seven days (here depicted: 3dpi). (B) Infection cushion formation is a process partially regulated by Nox complexes in *B.* *cinerea*. Agar plugs were set on onion epidermal layers and inoculated overnight. Just before microscopy, the fungal hyphae were stained by lactophenolblue. Scale bar = 10 µm. (C) Nox complex members produce different amount of ROS visualized by the TRD kit. Spores were grown in a microtiter plate for 12-16 h. Just before microscopy the detection agent for the visualization of ROS was added. Monitoring took place in a Tecan Saphire with 3 × 3 reads. Replicates displayed similar results. (D) BcNoxB is not involved in the formation of sclerotia. Previous results has revealed an effect of BcNoxB on the production of the perennial structures. However a more detailed characterization with new generated mutants showed that they still formed wild type like sclerotia [45]. Agar plugs were incubated on CM in constant darkness for at least 14 days.
