# Supplementary material for: Update on Nox function, site of action and regulation in Botrytis cinerea
Source: Fungal Biol Biotechnol. 2016 Oct 7;3:8. doi: 10.1186/s40694-016-0026-6 (PMC5611593; doi:10.1186/s40694-016-0026-6)
Supplement: Supplementary file 1 — Additional file 1: Table S1. Strains used in this work. [file 40694_2016_26_MOESM1_ESM.pdf]

S1

**Strain**
Δ*bcnoxA*
Δ*bcnoxA-C*
Δ*bcnoxA::bcnoxA_w/o* SP
B05.10 + roGFP2_NoxA
B05.10 + NoxA_roGFP2
Δ*bcnoxA:C1*
Δ*bcnoxA:C2*
Δ*bcnoxA:C3*
Δ*bcnoxB*
Δ*bcnoxB:C1*
Δ*bcnoxB:C2*
Δ*bcnoxB:C3*
Δ*bcnoxB::bcnoxB_HDEL*
Δ*bcnoxD*
Δ*bcnoxD::bcnoxD_HDEL*

**Genotype**
B05.10 Δ*bcnoxA::nat1*
B05.10 Δ*bcnoxA (nat)_bcnoxA_gfp (hph)*
Δ*bcnoxA (nat)::bcnoxA_w/o* SP *(hyg) in bcniiA*
B05.10, *roGFP2:bcnoxA* in *bcniaD*
B05.10, *bcnoxA:roGFP2* in *bcniiA*
Δ*bcnoxA (nat1)_PnoxB_bcnoxB_1-129_bcnoxA in bcniiA*
Δ*bcnoxA (nat1)_PnoxB_bcnoxA in bcniiA*
Δ*bcnoxA (nat1)_PnoxA_bcnoxB in bcniiA*
B05.10 Δ*bcnoxB::hph*
Δ*bcnoxB (hph)_PnoxB_bcnoxB_1-129_bcnoxA in bcniiA*
Δ*bcnoxB (hph)_PnoxB_bcnoxA in bcniiA*
Δ*bcnoxB (hph)_PnoxA_bcnoxB in bcniiA*
Δ*bcnoxB (hph)_bcnoxB_HDEL (nat) in bcniaD*
B05.10 Δ*bcnoxD::hph*
Δ*bcnoxD (hph)_bcNoxD_HDEL (nat) in bcniaD*

Description
Deletion of *bcnoxA*
Complementation of Δ*bcnoxA* with *bcnoxA_gfp*
Complementation of Δ*bcnoxA* with *bcnoxA* without the putative signalpeptide (AA 1-41)
Wild type with integration of *bcnoxA* fused to *roGFP2* at the 5´end; integration in *bcniaD* locus
Wild type with integration of *bcnoxA* fused to *roGFP2* at the 3´end; integration in *bcniiA* locus
Complementation of *bcnoxA* with *bcnoxA* fused to the first 129 aa of *bcnoxB* under the control of the *bcnoxB*
Complementation of *bcnoxA* with *bcnoxA* under the control of the *bcnoxB* promotor
Complementation of *bcnoxA* with *bcnoxB* under the control of the *bcnoxA* promotor
Deletion of *bcnoxB*
Complementation of *bcnoxB* with *bcnoxA* fused to the first 129 aa of *bcnoxB* under the control of the *bcnoxB*
Complementation of *bcnoxB* with *bcnoxA* under the control of the *bcnoxB* promotor
Complementation of *bcnoxB* with *bcnoxB* under the control of the *bcnoxA* promotor
Complementation of *bcnoxB* with *bcnoxB* fused to the HDEL motif for ER retention
Deletion of *bcnoxD*
Complementation of *bcnoxD* with *bcnoxD* fused to the HDEL motif for ER retention

Reference
Segmüller et al., 2008
Siegmund et al. 2013
This study
This study
This study
This study
This study
This study
Segmüller et al., 2008
This study
This study
This study
This study
Siegmund et al., 2015
This study
