# Supplementary material for: Update on Nox function, site of action and regulation in Botrytis cinerea
Source: Fungal Biol Biotechnol. 2016 Oct 7;3:8. doi: 10.1186/s40694-016-0026-6 (PMC5611593; doi:10.1186/s40694-016-0026-6)
Supplement: Supplementary file 2 — Additional file 2: Table S2. Oligonucleotides used in this work. [file 40694_2016_26_MOESM2_ESM.pdf]

S2

Nr.	Primer	Sequence 5'--> 3'	Purpose
1	BcnoxA_TglucR	CATACATCTTATCTACATACGCTAGAAATGTTC TTTCCAAAACG	Crosscomplementation
2	BcnoxA_NoxBN_R	CTTCAAAAACTGTACAGCACCCATCGTCAATTC CCTCGGCATTTCCGC	Crosscomplementation
3	bcnoxBProm_PtrpC_F	GCCCAAAAAATGCTCCTTCAATATCGTGTGGCG AGGGGGATTTGG	Crosscomplementation
4	noxA_PnoxB_R	CTTCAAAAACTGTACAGCACCCATCTTAAACGTt gCGACCTGGAATCG	Crosscomplementation
5	BcnoxB_TglucR	CATACATCTTATCTACATACGTTAGAAATTTTCT TTGCCCCAGC	Crosscomplementation
6	noxB_PnoxA_R	CTGTCATAAGCCTTTTCAGACATCTTCTCTATAT CCAAATATCCC	Crosscomplementation
7	bcnoxAprom_PtrpC_F	GCCCAAAAAATGCTCCTTCAATATCGACGACCC GGGCTCGTGCATAAG	Crosscomplementation
8	Ro1	CTCCATCACATCACAATCGATCCAAATGGTGAG CAAGGGCGAGGAGCTG	roGFP2_NoxA Construction (oliC_roGFP2)
9	Ro2	CCATCACATCACAATCGATCCAAATGGGTGCTG TACAGTTTTTGAAGG	NoxA_roGFP2 Construction (oliC_NoxA)
10	Ro3	CAGCTCCTCGCCCTTGCTCACCATGAAATGTTCT TTCCAAAACG	NoxA_roGFP2 Construction
11	Ro4	CGTTTTGGAAAGAACATTTCATGGTGAGCAAGG GCGAGGAGCTG	NoxA_roGFP2 Construction
12	Ro5	CATACATCTTATCTACATACGTTACTTGTACAGC TCGTCCATGCC	NoxA_roGFP2 Construction (roGFP2_Tgluc)
13	Ro6	CATACATCTTATCTACATACGCTAGAAATGTTC TTTCCAAAACG	roGFP2_NoxA Construction
14	Ro7	CGGCATGGACGAGCTGTACAAGATGGGTGCTGT ACAGTTTTTGAAGG	roGFP2_NoxA Construction
15	Ro8	CCTTCAAAAACTGTACAGCACCCATCTTGTACA GCTCGTCCATGCCG	roGFP2_NoxA Construction
16	Nox1	GGGAATGGATGAACTTTACAAAATGAGTATGGA TATCGAGAGGTGCC	NoxA_w/o signal peptide Construction
17	bcnoxA_F1	ATGGGTGCTGTACAGTTTTTGAAG	Crosscomplementation
18	bcnoxB_F1	ATGTCTGAAAAGGCTTATG	Crosscomplementation
19	hph for	GTCGGAGACAGAAGATGATATTGAAGGAGC	hph cassette
20	hph rev	GTTGGAGATTTCAGTAACGTTAAGTGGAT	hph cassette
21	BcniaD-HIbF/hi5F	CGCATATCAGCATATCGAGATGTCC	dia PCR niaD
22	BcniaD-HIaR/hi3R	GAGTACCCATCCGATGGAGTTGTTG	dia PCR niaD
23	BcniaD-WT-F	GCCACAGACTCCGCCAGATTCTAATG	dia PCR niaD
24	BcniaD-WT-R	CAACCATTTCACGCTGCGACCACC	dia PCR niaD
25	BcniiA-hi5F	GCGGGGTATGGCAGCATGAGTG	dia PCR niiA
26	BcniiA-hi3R	CTTATAGCAAGCGCGATGTGTATC	dia PCR niiA
27	BcniiA-WT-F	GGTTGAGGTGGTGGAAGATTTG	dia PCR niiA
28	BcniiA-WT-R	CGACCACCAAGCCTCCAGCATC	dia PCR niiA
29	NoxD_GFP 5F	CCATCACATCACAATCGATCCAACCATGGGAAG ACTTATCAAGAACC	NoxD_HDEL generation
30	Nox103	CTTACCTCACCCTTGGAAACCATAAGTTCATCA TGAACTTTCCCCGTGGCGGATCTCCG	NoxD_HDEL generation
31	BcniaD_SacII_5F	GTAACGCCAGGGTTTTCCCAGTCACGACGCCGC GGGTGAATGGGATTCATTGTTTATTTC	NoxB_HDEL generation
32	Nox96	CTAATCATACATCTTATCTACATACGTTAAAGTT CATCATGGAAATTTTCTTTGCCCCAG	NoxB_HDEL generation
33	Tgluc_HDEL_noxB-R	TCTTATCTACATACGTTAAAGTTCATCATGGAA ATTTTCTTTGCCCCAG	NoxB_HDEL generation
34	BcnoxB_OGFP_F	GGGAATGGATGAACTTTACAAAATGTCTGAAAA GGCTTATGACAG	NoxB_HDEL generation
35	noxA_diaF1	GCTCAGATCATTCTTCTTTG	NoxA diagnostic PCR
36	noxA_diaR1	CGTAATGACCTCCTGTCAGAATG	NoxA diagnostic PCR
37	Hph-hiF	GTCTGGACCGATGGCTGTGTAGAAG	diagnostic PCR
38	hph-hiR	GACAGACGTCGCGGTGAGTTCAG	diagnostic PCR
39	Nat1-hiF	CGGCGAGCAGGCGCTCTACATGAGC	diagnostic PCR
40	nat1-hiR	GGTAAGCCGTGTCGTCAAGAG	diagnostic PCR
