# Supplementary material for: Update on Nox function, site of action and regulation in Botrytis cinerea
Source: Fungal Biol Biotechnol. 2016 Oct 7;3:8. doi: 10.1186/s40694-016-0026-6 (PMC5611593; doi:10.1186/s40694-016-0026-6)
Supplement: Supplementary file 3 — Additional file 3: Figure S3. Bioinformatic analysis of BcNoxA and BcNoxB. (A) Protein sequence of BcNoxA with the highlighted putative signal peptide. The prediction was accomplished using the SOSUIsignal algorithm. (B) Alignment of BcNoxA and BcNoxB using the Clustal Ω algorithm. The N-terminal elongation of NoxB was used in the cross complementation experiments. Asterisk highlight identical amino acids, while colons are indicating amino acids with similar characteristics. Dots refer to a faint resemblance. [file 40694_2016_26_MOESM3_ESM.pdf]

A

Putative signal peptide (41 aa)

BcNoxA (553 aa)

MGAVQFLKEQTRGTKLLFNFLFHGFHVGLFALGWWKQASDPRLAGLNSLTFSVWISRGAG
LVLSVDVALILLPMCRNILRYIRPKIKFLPLDESQWFHRQVAYSLLFWTIFHVAAHYVNF
FNVEKTQIRPLTAIQIHYTEAGGITGHIMLLCMMLMYTTAHAKIRQQSFETFWYTHHLFI
PFLLGMYTHATGCFVRDTADPFSPFDGENFWGHCLGYEGWRWELWGGGLYFLERVYREIR
SRRETQIVRVVRHPYDAMEIQFRKPSMKYKAGQWLFLNVPSVSREQWHPFTITSCPFDPY
ISIHIRQVGDFTRTLGDALGAGAAQAKLYDGVDPNGMYEVALENGQKMPDIRIDGPYGAP
AEDVFENEVAVLIGTGIGVTPWASILKNIWHLRHGTNPPERLRRVEFIWVCKDTTSFEWF
QVLLSSLEAQSQEAAGQPGNDGQEFLRIHTYLTQKLDIDTAQNIVLNSVGADVDPLTELK
SRTNFGRPDFTKLFEGMRDGIMDKTYMSGLEGDFKTNVGVYFCGPNVAARSIKKACKNAT
TRDVNFSFWKEHF

B

N-terminal elongation of BcNoxB (43aa/129bp)

```
NoxB        MSEKAYDRMSTGSVRVSERSRWPPLTRMLMSGEMTAEMPRELTMREKFDRWMVNEGYRRF
NoxA        -------------------------------------------MGAVQFLKEQTRGTKLL
                                                       *         ..* : :

NoxB        FVFVFALLHVMVFTFGFLNYQLKDSFGIARSTFTVTYAIARSAALTLHFDVAMILFPVCR
NoxA        FNFLFHGFHVGLFALGWWKQASDPRL-AGLNSLTFSVWISRGAGLVLSVDVALILLPMCR
            * *:*  :** :*::*: :   .  :  . .::*.:  *:*.*.*.* .***:**:*:**

NoxB        TLISLARQTPLNGIVQFDKNITFHMLTAWSIVFFSWVHTIAHWNNFAQISAKNNLGFGGF
NoxA        NILRYIRP--KIKFLPLDESQWFHRQVAYSLLFWTIFHVAAHYVNFFNVEKTQIRPLT-A
            .::    *      :: :*:.  **   .*:*::*:: .*. **: ** ::. .:    :

NoxB        LLANFVSGPGWTGYVMLIALMAMAITSYEKYRRANFERFWYTHHFFVIFFVFWSVHGAFC
NoxA        IQIHYTEAGGITGHIMLLCMMLMYTTAHAKIRQQSFETFWYTHHLFIPFLLGMYTHATGC
            :  .:... * **::**:.:* *  *:: * *: .** ******:*: *::    .*.: *

NoxB        MIQPDTAPYCVSVGTSAIG-----VFWQYWMYGGYIYLAERIAREIRGKHKTYVSKVVQH
NoxA        FVRDTADPFSPFDGENFWGHCLGYEGWRWELWGGGLYFLERVYREIRSRRETQIVRVVRH
            :::  : *:.    *  .  *        *:: ::** :*: **: ****.:::* : :**:*

NoxB        PSNVCEIQIKKENTKTRAGQYIFFCCPEVSIYQYHPFTLTSAPEEDYISIHIRMVGDFTR
NoxA        PYDAMEIQFRKPSMKYKAGQWLFLNVPSVSREQWHPFTITSCPFDPYISIHIRQVGDFTR
            * :. ***::* . * :***::*:  *.**  *:****:**.* : ******* ******

NoxB        AVGKALGCEFDKPKDGKGGSQVVGVNQGSPGSDGADSAIRRILPRVYVDGPFGSASEDVF
NoxA        TLGDALGAGAAQAKLYDGVD--------PNGMYEVALENGQKMPDIRIDGPYGAPAEDVF
            ::*.***.   : *  .* .          *   .     : :* : :***:*: :****

NoxB        KFEVAMLCGAGIGVTPFASILKSIWYRMNYPQKKTRLGKVYFFWICRDFGSFEWFRSLLL
NoxA        ENEVAVLIGTGIGVTPWASILKNIWHLRHGTNPPERLRRVEFIWVCKDTTSFEWFQVLLS
            : ***:* *:******:*****.**:    .    :   ** :* *:*:*:*  *****: **

NoxB        AIEAQDM----------DNRIEIHTYLTAKIKVDDATNIMINDANADRDAITGLRAPTNF
NoxA        SLEAQSQEAAGQPGNDGQEFLRIHTYLTQKLDIDTAQNIVLNSVGADVDPLTELKSRTNF
            ::***.          :: :.****** *:.:* * **::*.. ** * :* *:: ***

NoxB        GRPNWDMIFKSVRKI-------------HSPSEAGVFFCGPKVLGSQLHIKCNMYSEPGFN
NoxA        GRPDFTKLFEGMRDGIMDKTYMSGLEGDFKTNVGVYFCGPNVAARSIKKACKNATTRDVN
            ***::  :*:.:*.             .  ::.**:****:* . .::  *:  :   .*

NoxB        FCWGKENF
NoxA        FSFWKEHF
            *.: **.*
```
