# Supplementary material for: Update on Nox function, site of action and regulation in Botrytis cinerea
Source: Fungal Biol Biotechnol. 2016 Oct 7;3:8. doi: 10.1186/s40694-016-0026-6 (PMC5611593; doi:10.1186/s40694-016-0026-6)
Supplement: Supplementary file 4 — Additional file 4: Figure S4. Transcriptional regulation is not important for NoxB function. Three complementation constructs (C1–C3) were generated and transformed in ΔbcnoxB. Integration took place into the bcniiA locus (Table S1). (A) Wild type like spore-mediated infection is restored in ΔbcnoxB:C3. Primary bean leaves were infected with 7.5 µl of conidial suspension (105 conidia/ml). Lesion diameters were measured and statistically evaluated (3 bean plants/strain). (B) ΔbcnoxB, ΔbcnoxB:C1 and ΔbcnoxB:C2 are deficient in forming functional appressoria. Onion epidermal layers were infected with drops of a conidial suspension (105 conidia/ml). Just before microscopy the fungal hyphae were stained with lactophenol blue. (C) Only ΔbcnoxB:C3 produces the same amount of conidiospores as the wild type. Spores were harvested from CM plates with 10 ml H2O and quantified in replicates. (D) Plate assays for the determination of the stress resistance display identical results for the wild type and the strain ΔbcnoxB:C3. Agar plugs were placed on CM agar and medium supplemented with H2O2 (10 mM) or menadione (500 µM). Monitoring was accomplished for seven days. (E) Only ΔbcnoxB:C3 produces wild type like levels of ROS in microplate assays with the TRD kit. Spores were grown in a microtiter plate for 12-16 h. Just before microscopy the detection agent for the visualization of ROS was added. Monitoring took place in a Tecan Saphire with 3 × 3 reads. Replicates displayed similar results. Scale bars = 10 µm. [file 40694_2016_26_MOESM4_ESM.pdf]

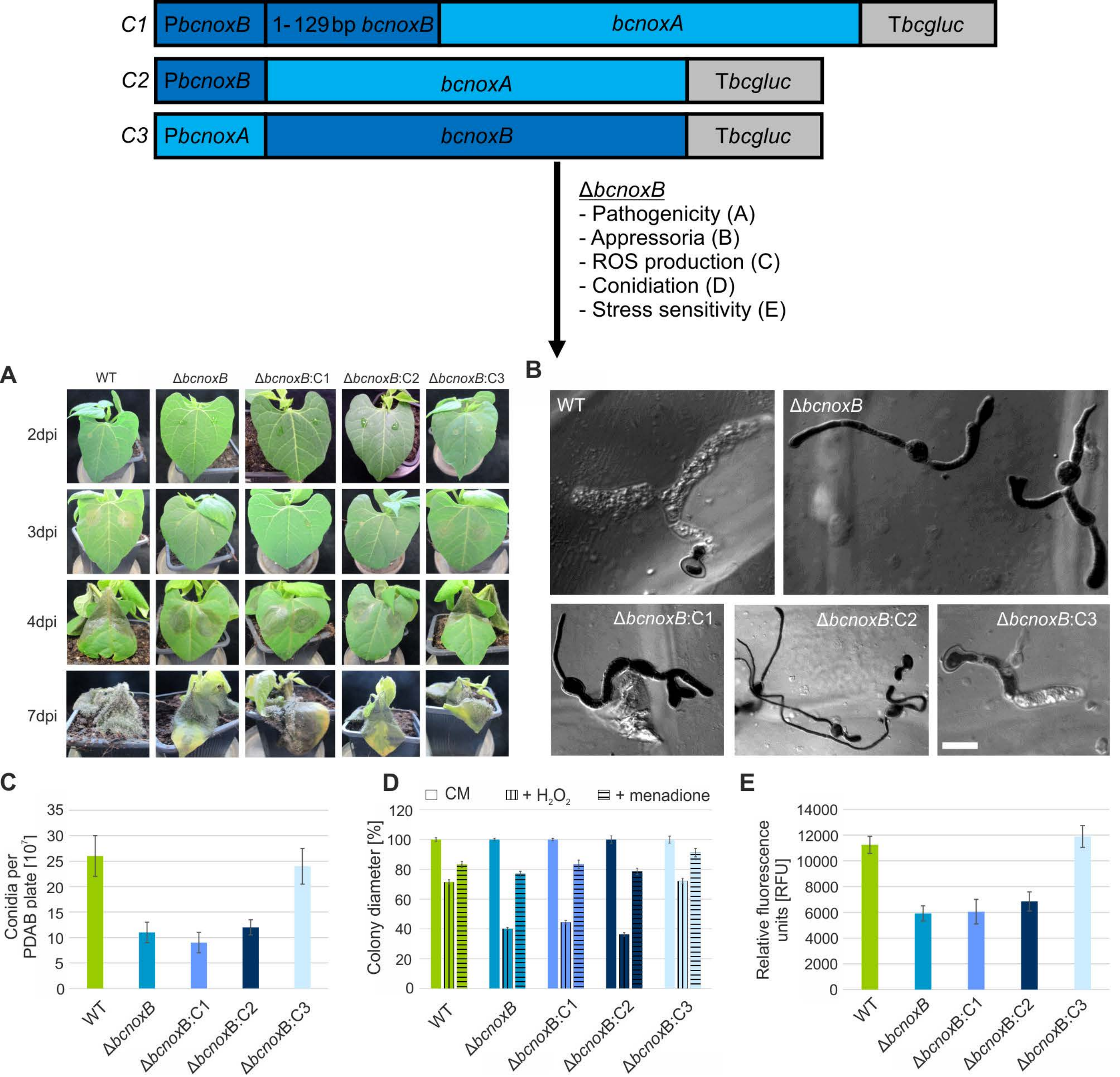

C1
PbcnoxB
1-129 bp bcnoxB
bcnoxA
Tbcgluc
C2
PbcnoxB
bcnoxA
Tbcgluc
C3
PbcnoxA
bcnoxB
Tbcgluc
ΔbcnoxB
- Pathogenicity (A)
- Appressoria (B)
- ROS production (C)
- Conidiation (D)
- Stress sensitivity (E)
A
WT
ΔbcnoxB
ΔbcnoxB:C1
ΔbcnoxB:C2
ΔbcnoxB:C3
2dpi
3dpi
4dpi
7dpi
B
WT
ΔbcnoxB
ΔbcnoxB:C1
ΔbcnoxB:C2
ΔbcnoxB:C3
C
Conidia per PDAB plate [10^7]
0 5 10 15 20 25 30 35
WT
ΔbcnoxB
ΔbcnoxB:C1
ΔbcnoxB:C2
ΔbcnoxB:C3
D
CM
+ H2O2
+ menadione
Colony diameter [%]
0 20 40 60 80 100 120
WT
ΔbcnoxB
ΔbcnoxB:C1
ΔbcnoxB:C2
ΔbcnoxB:C3
E
Relative fluorescence units [RFU]
0 2000 4000 6000 8000 10000 12000 14000
WT
ΔbcnoxB
ΔbcnoxB:C1
ΔbcnoxB:C2
ΔbcnoxB:C3
