# Supplementary figures and images for: Update on Nox function, site of action and regulation in Botrytis cinerea
Source: Fungal Biol Biotechnol. 2016 Oct 7;3:8. doi: 10.1186/s40694-016-0026-6 (PMC5611593; doi:10.1186/s40694-016-0026-6)

A

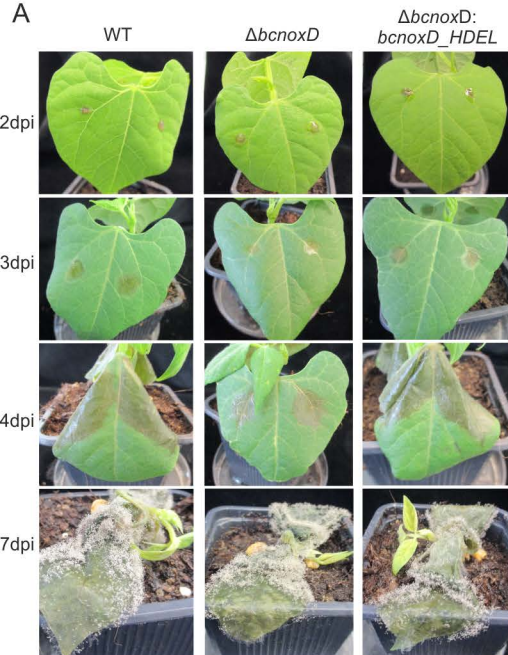

B

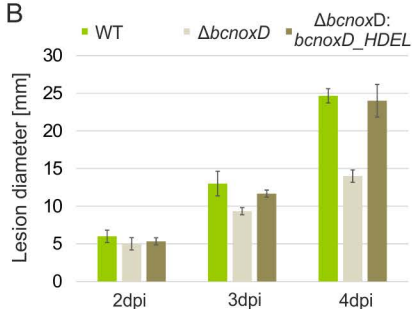

C

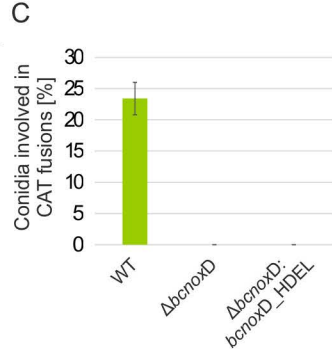

D

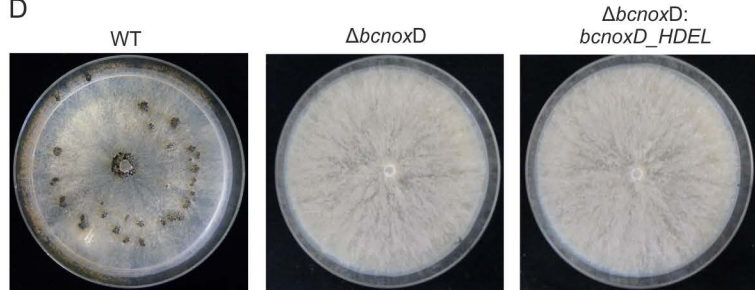

E

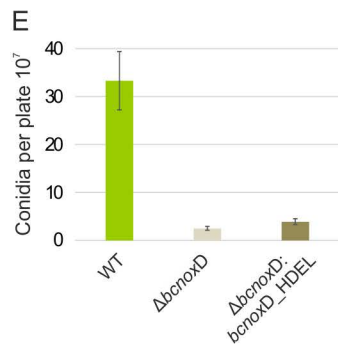

F

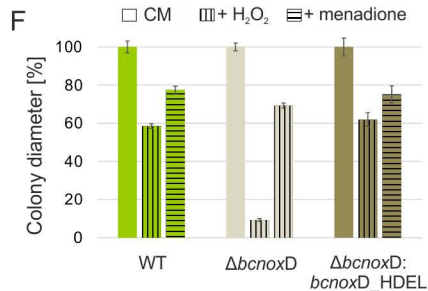

G

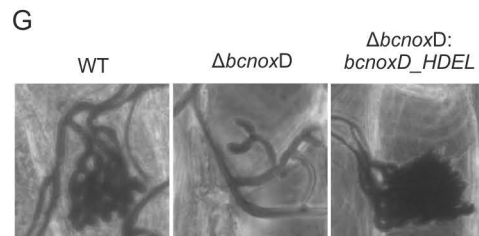

Supplement: Supplementary file 5 — Additional file 5: Figure S5. BcNoxD_HDEL resembles the phenotype of BcNoxA_HDEL and has functions inside and outside the ER. ΔbcnoxD was complemented with an ER locked allele of bcnoxD. The construct was integrated into the bcniaD locus (Table S1). (A/B) The infection defect of ΔbcnoxD is fully restored by the ER locked allele of BcNoxD. Bean leaves were inoculated with conidiospores (105 conidia/ml) and monitored until the tissue was fully macerated. Lesion diameters were measured and statistically evaluated (3 bean plants/strain). (C) The ER bound allele of NoxD is not sufficient to restore the formation of hyphal fusions. Conidia were incubated on minimal medium for 18 h. Hyphal fusions were detected microscopically and evaluated statistically (300 spores each). (D) Sclerotia production is restored in the strain ΔbcnoxA:bcnoxD_HDEL. Agar plugs were incubated on CM in constant darkness for at least 14. (E) ΔbcnoxA:bcnoxD_HDEL displayed reduced levels of conidiospores. Spores were washed down from CM plates with 10 ml H2O and quantified in three replicates. (F) Stress resistance was tested on CM agar in comparison to selective media supplemented with H2O2 (10 mM) or menadione (500 µM) for seven days. Colony diameters were measured (here depicted: 3 dpi). The ER locked allele mediates resistance against oxidative stress conditions. (G) Infection cushion formation is restored in ΔbcnoxA:bcnoxD_HDEL. Onion epidermal layers were inoculated with agar plugs of the respective strains. Staining of fungal hyphae was performed with lactophenol blue just before microscopy. [file 40694_2016_26_MOESM5_ESM.pdf]

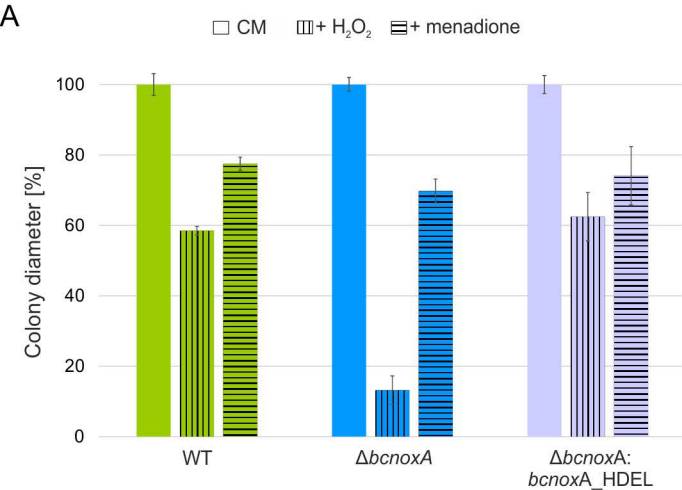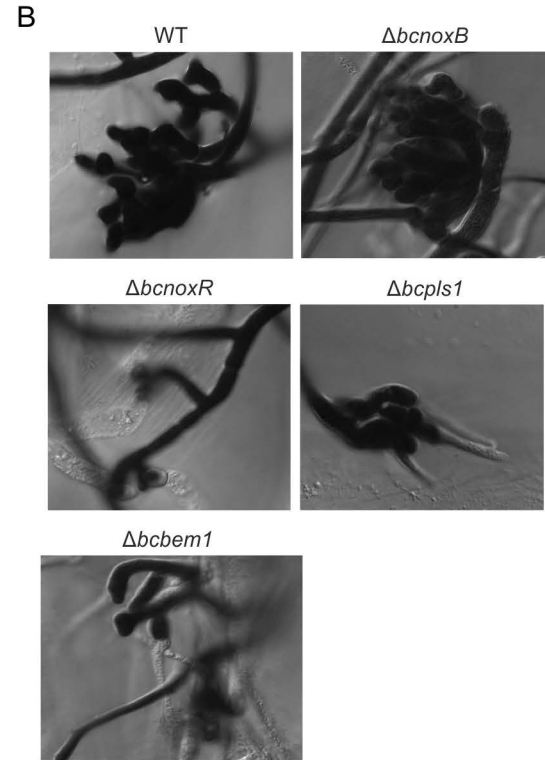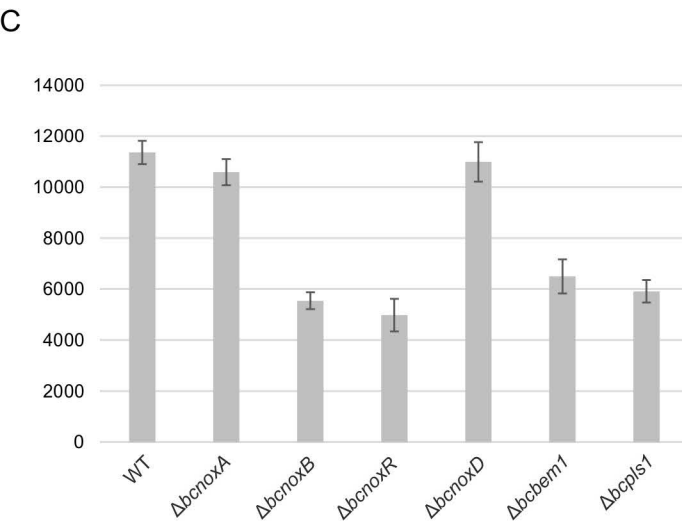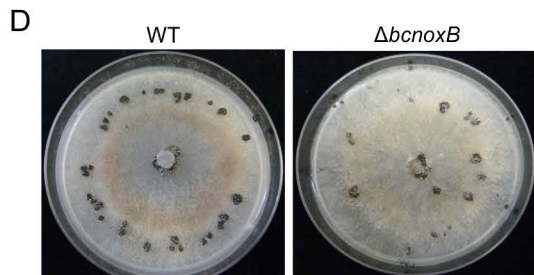

Supplement: Supplementary file 6 — Additional file 6: Figure S6. Differentiation processes affected by members of Nox complexes in B. cinerea (A) BcNoxA mediates stress resistance when hooked to the ER membrane. ΔbcnoxA was complemented with an ER locked allele of bcnoxA and tested for stress sensitivity against oxidative stress agents such as H2O2 (10 mM) or menadione (500 µM). Stress resistance is fully restored by the ER locked allele of BcNoxA. Colony diameters were measured for seven days (here depicted: 3dpi). (B) Infection cushion formation is a process partially regulated by Nox complexes in B. cinerea. Agar plugs were set on onion epidermal layers and inoculated overnight. Just before microscopy, the fungal hyphae were stained by lactophenolblue. Scale bar = 10 µm. (C) Nox complex members produce different amount of ROS visualized by the TRD kit. Spores were grown in a microtiter plate for 12-16 h. Just before microscopy the detection agent for the visualization of ROS was added. Monitoring took place in a Tecan Saphire with 3 × 3 reads. Replicates displayed similar results. (D) BcNoxB is not involved in the formation of sclerotia. Previous results has revealed an effect of BcNoxB on the production of the perennial structures. However a more detailed characterization with new generated mutants showed that they still formed wild type like sclerotia [45]. Agar plugs were incubated on CM in constant darkness for at least 14 days. [file 40694_2016_26_MOESM6_ESM.pdf]
